# Impact of Temporal Variation on Design and Analysis of Mouse Knockout Phenotyping Studies

**DOI:** 10.1371/journal.pone.0111239

**Published:** 2014-10-24

**Authors:** Natasha A. Karp, Anneliese O. Speak, Jacqueline K. White, David J. Adams, Martin Hrabé de Angelis, Yann Hérault, Richard F. Mott

**Affiliations:** 1 Wellcome Trust Sanger Institute, Hinxton, Cambridgeshire, United Kingdom; 2 German Mouse Clinic - Institute of Experimental Genetics, Helmholtz Zentrum München, Neuherberg, Germany; 3 Experimental Genetics, Technische Universität München, Freising-Weihenstephan, Germany; 4 German Center for Diabetes Research, Neuherberg, Germany; 5 Institut Clinique de la Souris, Université de Strasbourg, Illkirch, France; 6 The Wellcome Trust Centre for Human Genetics, University of Oxford, Oxford, Oxfordshire, United Kingdom; University of Aberystwyth, United Kingdom

## Abstract

A significant challenge facing high-throughput phenotyping of *in-vivo* knockout mice is ensuring phenotype calls are robust and reliable. Central to this problem is selecting an appropriate statistical analysis that models both the experimental design (the workflow and the way control mice are selected for comparison with knockout animals) and the sources of variation. Recently we proposed a mixed model suitable for small batch-oriented studies, where controls are not phenotyped concurrently with mutants. Here we evaluate this method both for its sensitivity to detect phenotypic effects and to control false positives, across a range of workflows used at mouse phenotyping centers. We found the sensitivity and control of false positives depend on the workflow. We show that the phenotypes in control mice fluctuate unexpectedly between batches and this can cause the false positive rate of phenotype calls to be inflated when only a small number of batches are tested, when the effect of knockout becomes confounded with temporal fluctuations in control mice. This effect was observed in both behavioural and physiological assays. Based on this analysis, we recommend two approaches (workflow and accompanying control strategy) and associated analyses, which would be robust, for use in high-throughput phenotyping pipelines. Our results show the importance in modelling all sources of variability in high-throughput phenotyping studies.

## Introduction

Phenotyping, the process by which an organism’s observable characteristics are measured, is an essential component of biological research. However, there is need for improvements in phenotyping methodology to improve reproducibility and reduce the sensitivity of assays to the environment [1]–[3].

The mouse is the premier model organism for understanding gene function in development and disease. The International Mouse Phenotyping Consortium (IMPC) [4] aims to phenotype knockouts for all mouse protein coding genes, building on the large collection of targeted alleles in C57BL/6N embryonic stem cells available from the International Knockout Mouse Consortium [5]–[7]. Currently ten centers, are screening mutant mouse strains with a series of standardised tests carried at specific ages [4]. To do this, each centre developed and implemented its own phenotyping pipeline reflecting its local facilities and constraints. The IMPC has developed a central resource (www.mousephenotype.org) to disseminate and share all animal, experimental and phenotypic data from every mutant line analysed. These data can be mined to determine which factors influence phenotyping experiments and whether different workflows produce comparable results.

Here, a phenotyping pipeline means a well-defined sequence of phenotyping procedures carried out at specific ages. To date, standardisation has focused on the experimental methods by which data were collected [8], [9]. However the workflow - the practical implementation of a pipeline - varies from centre to centre. Each centre’s workflow is a balance of resources, other goals (e.g. allowing for additional phenotyping depending on earlier results) and throughput requirements. Differences in the number and frequency of controls, whether knockout animals are phenotyped at one time or in multiple batches, and blinding methodologies are the most important variables. Batch (defined here as those readings collected on a particular day) is a significant source of variation [10] and consequently how a pipeline is implemented (the workflow) is critical to how data from a pipeline should be analysed.

For the purposes of this study, workflows vary in two important ways. First, whether there are concurrent controls for each assay day (batch). The use of concurrent controls significantly reduces the capacity of the pipeline because far more controls are phenotyped. Second, the breeding strategy implemented affects the generation of knockout animals with the appropriate age range. The breeding strategy and resulting batch size depends on capacity of vivarium, budget, fertility, fecundity, and viability. In consequence, controls are not necessarily phenotyped concurrently with each knockout line nor are the knockout animals all phenotyped in one batch. In the presence of temporal variation, where the mice in the same batch are likely to be more similar than those from different batches, these issues make phenotyping a challenge, particularly as only seven mice per knockout strain per sex are phenotyped. With variation in workflow across institutes, this raises the question as to how data should be analysed to reliably identify phenotypes for the community.

There are two published analysis methodologies for quantitate traits that try to account for these issues. In the reference range (RR) methodology, a mouse knockout is classed as having a phenotype of interest if the majority of the knockout animals lay outside the range of variation observed in the controls [11]. This is a conservative, non-quantitative method that has a non-constant significance levels and restricts downstream analysis of the data [10]. With the RR methodology implemented at Wellcome Trust Sanger Institute (WTSI), the probability of observing a significant phenotype by chance is about 6×10^−6^ per sex per tested phenotype, and the chance that either sex is significant is about 1.2×10^−5^ per phenotype. The disadvantage of the RR is that it does not take into account batch variation, and as we show below, it is thereby possible to make false positive RR calls despite its apparently stringent significance threshold. Recently, we demonstrated that a mixed model (MM) methodology in which temporal variation is modelled gave significantly improvements [10]. The methodology is implemented in the PhenStat R package [12]. PhenStat starts with a model that includes fixed effects for sex, genotype, genotype-by-sex interaction and optionally weight, and a random effect for batch. It tests the significance of each of these terms in order to optimise the assessment of the genotype effect. The genotype-by-sex effect is also tested to determine whether there is a sexual dimorphic knockout effect.

In this study we investigate the impact of temporal variation and workflow differences on our ability to call phenotypes. To do this, we constructed datasets sampled from real control data from the WTSI MGP Select pipeline to mimic various workflows. To investigate false positive rates (FPR) we relabelled a subset of controls as knockout mice and then compared them to the remaining controls. Thus at a nominal *p*% significance threshold there should be *p*% significant calls if the statistical test is accurate when the null hypothesis is true. We consider data from five assays to ensure the findings are representative. The assays studied included the open field behavioural assay, three blood parameters (peripheral blood leukocytes, haematology and clinical chemistry) and Dual-energy X-ray absorptiometry (DEXA) that assesses body composition. We also investigated data from other phenotyping institutes. We show our ability to call phenotypes accurately and sensitivity depends on the workflow. We observed the FPR was inflated in certain workflows, so we constructed and tested further simulated data that perfectly met the mathematical assumptions underlying the mixed model. Our analysis demonstrates that the inflated FPR is associated with the distribution of control data, combined with the workflow characteristics. We also show that unexpectedly large batch-to-batch fluctuations in the control data, cause false positive significant phenotype calls when mice are phenotyped across only a few batches. Finally we identify workflow implementations and control strategies that are robust to the presence of environment fluctuations.

## Methods

### Ethics

The care and use of mice in the WTSI study was carried out in accordance with UK Home Office regulations, UK Animals (Scientific Procedures) Act of 1986 under two UK Home Office licences which approved this work (80/2076 and 80/2485) which were reviewed regularly by the WTSI Ethical Review Committee. The care and use of mice at the German Mouse Clinic (permit: 55.2-1-54-2532) was in accordance of the German Animal Welfare Act and were approved by the Government of Upper Bavaria via the Regierung von Oberbayern committee. The care and use of mice at the Institut Clinique de la Souris were performed under protocols (2011-024a and 2011-024b) approved by the Com’Eth committee.

All efforts were made to minimize suffering by considerate housing and husbandry (see Methods S1 for details). Animal welfare was assessed routinely for all mice involved. Adult mice were killed by terminal anaesthesia followed by exsanguination and either cervical dislocation or removal of the heart.

### Datasets

For information on datasets; including the housing and husbandry, procedural methods for collection of data, and how data can be accessed please see Methods S1.

### Construction of artificial data

Artificial control data were constructed based on the current knowledge of having sex and temporal variation as dominate sources of variation and assumes that the temporal variation arises from a batch effect that is independent normally distributed. As these sources of variation are modelled within the mixed model, this data would meet the assumptions of the mixed model test perfectly. A script (Code S1) generated data by randomly sampling from a population with defined mean and standard deviation. Batch variation was simulated under the assumption it was normally distributed with mean zero and defined variance. Data were generated for fifteen dependent variables with 300 independent batches for different values of the population mean and standard deviation, and batch variance.

### Assessing the false positive rate (FPR)

Resampling studies were conducted to assess the FPR under the null hypothesis. Mice (7 males and 7 females) were selected from the control dataset without replacement and relabelled as knockout. Prior to combining the knockout mice back with the control mice dataset, the mice selected to be relabelled as knockout were removed from the control dataset. The resulting dataset were then examined statistically for statistically significant differences between control and fake knockout mice (phenodeviants), which would all be false positives. The mice were selected in a manner to mimic the various workflows used in high throughput pipelines, which depend on breeding strategy and pipeline operational issues (Table 1). Up to 500 datasets were constructed by selecting different mice based on the workflow rules. For certain workflows the number of distinct datasets that could be constructed is limited by the structure of the control data (number and size of batch). The numbers of datasets tested in each study are given in (Table S1).

**Table 1:** Description of workflow tested.

Workflow	Classification	Resampling strategy
Random	*multi-batch*	For each sex, 7 assay dates selected randomly and one mouse randomly selected from this.
Multi-Group	*multi-batch*	For each sex, 3 assay dates selected randomly. From one date, assay 3 mice, and 2 mice from the other dates.
OneBatch	*traditional*	7♂ and 7♀ mice selected from one assay date.
TwoBatch	*low-batch*	4♂ & 4♀ selected for an assay date, from the next sequential assay date 3♂ & 3♀.
ThreeBatch	*low-batch*	3♂ & 3♀ selected for an assay date, from the next sequential assay date 2♂ & 2♀ and then from the next sequential date 2♂ & 2♀.

### Assessing the statistical power

Simulated control data, comprising 300 batches with 7 males and 7 females in each date, were constructed based on the population characteristics estimated for the WTSI DEXA control dataset. The population characteristics were estimated by fitting a mixed model with sex and weight as a fixed effect and assay date as a random effect. From the model fitting, estimates were extracted for the male and female biological mean. The standard deviation estimated on the intercept equating the female data was taken as the biological standard deviation. To assess the impact of batch variability, datasets were constructed with varying amounts of batch variant (from 25 to 400% of the estimated biological standard deviation). Phenodeviant mice were constructed by sampling 7 male and 7 female animals from the simulated dataset, adding a defined signal and relabeling as knockout mice and assessed using PhenStat (model: without Weight). To assess sensitivity, the amount of signal added varied between 25 to 200% of the estimated standard deviation within a batch for a variable. This process was completed 500 times for each variable for each scenario. The study was repeated independently for each workflow studied (see Table 1).

### Identification of significant phenotypes

An iterative top down mixed modelling strategy was performed as described in [10] using PhenStat an R package [12] freely available from Bioconductor [13]. There are two possible start models, depending on whether weight is included as a factor (see Eq1. and Eq2.). The complexity of the model is limited by the low number of mice used in high throughput studies as such key fixed effects have been selected.
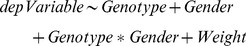
(1)

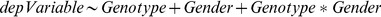
(2)


## Results

### Investigating reproducibility issues

**Figure 1:**
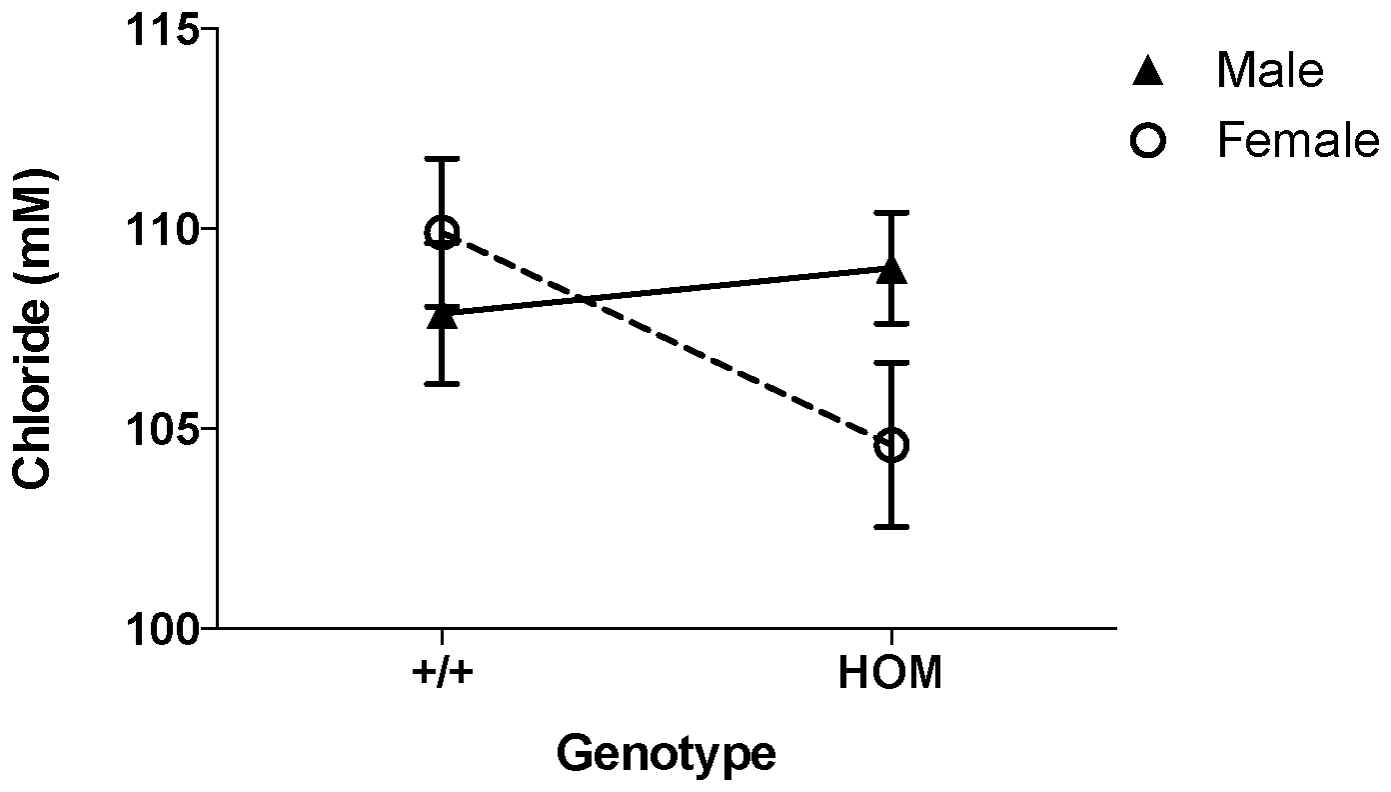
Example false positive call. Shown are chloride blood chemistry measures collected for *Expi^tm1a(KOMP)Wtsi^*/*Expi^tm1a(KOMP)Wtsi^* (HOM) and wildtype (+/+) mice at week 16 with the high throughput pipeline. The phenotype was classed as female specific effect (p value: 0.0016, Genotype by female effect quantified as −5.34±1.29 (se) with PhenStat MM without weight). Comparison based on 298 female and 264 male wildtype mice and 7 female and 6 male knockout mice.

**Figure 2:**
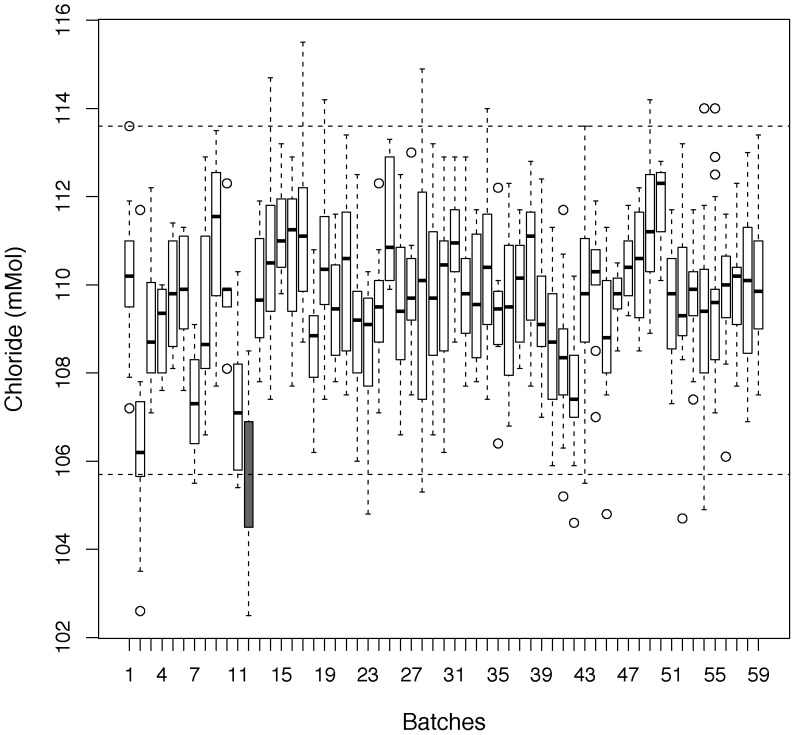
Variation of chloride readings with time. Shown are chloride blood chemistry measures collected at week 16 by batch for those collected within 2012 for both knockout and control mice collected on the WTSI MGP Select high throughput pipeline for the core strain B6N. The dotted lines indicate the 95% percentile values. The boxplot highlighted in black shows the date on which the *Expi^tm1a(KOMP)Wtsi^* female data were collected, and shows that all values collected on that date were low. Whilst these data points were low, the instruments daily QC checks were within the required boundaries.

At WTSI, we have found instances where phenotype calls that were significant according to either the RR and MM criteria were not reproducible. These cases often occurred when the 14 knockout mice were phenotyped across only four or fewer batches (*low-batch/traditional* workflow), The knockout *Expi^tm1a(KOMP)Wtsi^* is a typical example. Two clinical chemistry variables (sodium and chloride) appeared to be highly significant for the female mice both by the MM and the more conservative reference range (RR) methodology (Example shown in Figure 1). However these phenotype calls were not replicated during secondary phenotyping, despite the apparently large phenotypic effect which gave the secondary experiment high sensitivity (power = 0.957)(data not shown). Further investigation showed that all the knockout females were phenotyped on a single day without concurrent controls, and all data collected on that day were low compared to other days, but still within instrument quality control checks (Figure 2).

**Figure 3:**
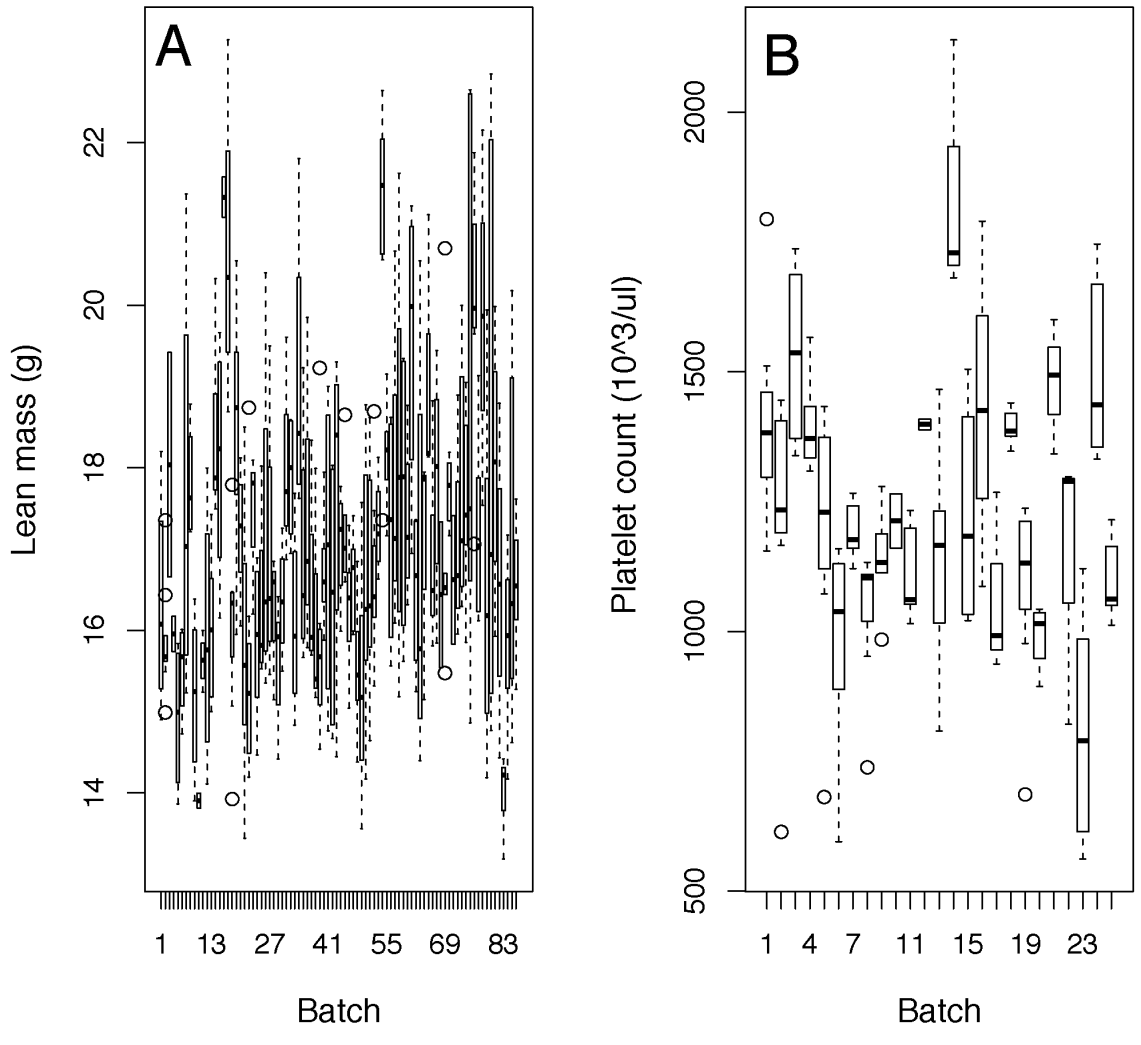
Example temporal variation in control data. Shown are box and whisker plots as function of batch (x-axis) A: Fat mass readings for Institut Clinique de la Souris IMPC pipeline control C57BL/6NTac male mice B: Platelet readings for the German Mouse clinic IMPC pipeline control C57BL/6NTac(USA) male mice.

This problem is not specific to the WTSI, as it is found in control data from two other independent high throughput programs at the German Mouse Clinic (GMC) and Institut Clinique de la Souris (ICS). Similar to WTSI control data, analysis of variance showed that on average batch accounted for a quarter of the variation (WTSI: 22.3±1.5%, GMC: 27.7±2.0%, ICS: 27.3±2.2% (mean ± standard error of the mean)), whilst sex accounted for around 10% (WTSI: 11.9±1.8%, GMC: 8.2±1.9%, ICS: 12.1±2.0% (mean ± standard error of the mean)). The contribution of weight depended on the assay (data not shown). An example of temporal batch variation is plotted in Figure 3.

### Using control data to mimic various workflows

To understand the variation to be expected under the null hypothesis that there is no phenotypic effect due to the knockout allele, we simulated a variety of different workflows, the details of which are described in Table 1 and Figure S1. We sampled phenotype data from control animals on different days to mimic a given workflow. Workflows can be grouped into three main categories based on how many batches of knockout animals are tested per line, which we call *traditional, multi-batch*, and *low-batch* workflows. The *traditional* workflow is a one batch design where all phenotype data for a given knockout and procedure are collected on one day, and typically with concurrent controls. In a *multi-batch* workflow, the knockout mice are phenotyped over a minimum of 5 days. In a *low-batch* workflow, the knockout mice are phenotyped over between one to four days. This includes a workflow where mice are phenotyped in two batches, one for each sex. In our simulations control mice were selected at random from the corresponding number of batches using real data from the WTSI MGP Select pipeline. We then assigned the sampled animals to knockout and control groups in order to mimic the given workflow. Thus, if our mixed-model statistical analysis is valid then we should expect to call *p*% of the simulations as significant at the *p*% level.

**Figure 4:**
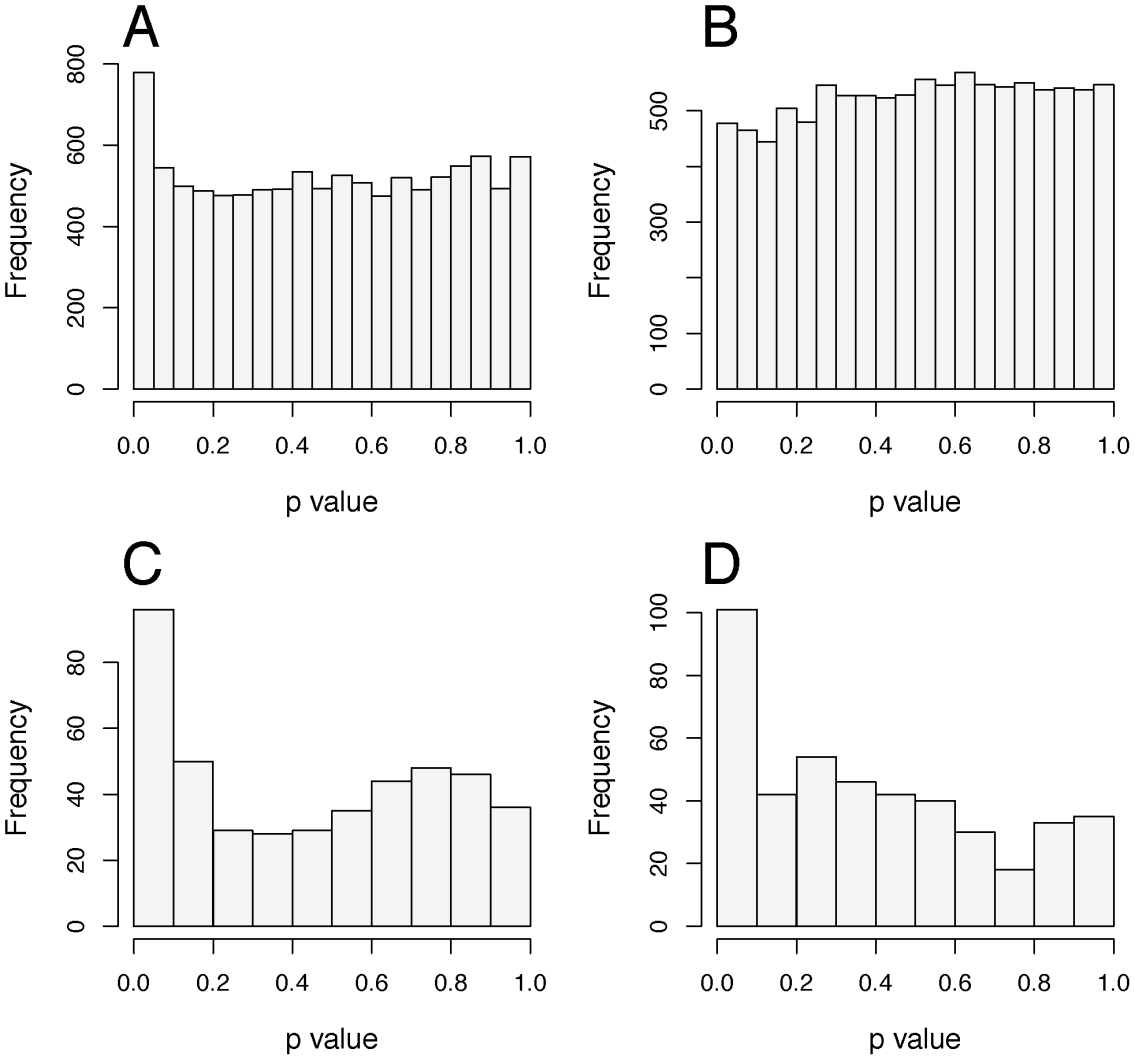
Variation in distribution of p-values with workflow. Example empirical distribution profiles for the resampling of the seven traits measured in the Dual-energy X-ray absorptiometry control data. A and B: Multi-Group workflow where A shows the test of genotype effect and B the test of genotype-by-sex effect. C and D: One batch per colony workflow where C shows the test of genotype effect and D the test of genotype-by-sex effect.

For some workflows we found the empirical distribution was close to the ideal, whilst others have an inflated FPR with a spike of low *p*-values (Figure 4 and Table S2). This workflow effect was independent of assay, trait or whether weight was included as a covariate (data not shown). It was also independent of the significance thresholds selected (Figure S2).

**Figure 5:**
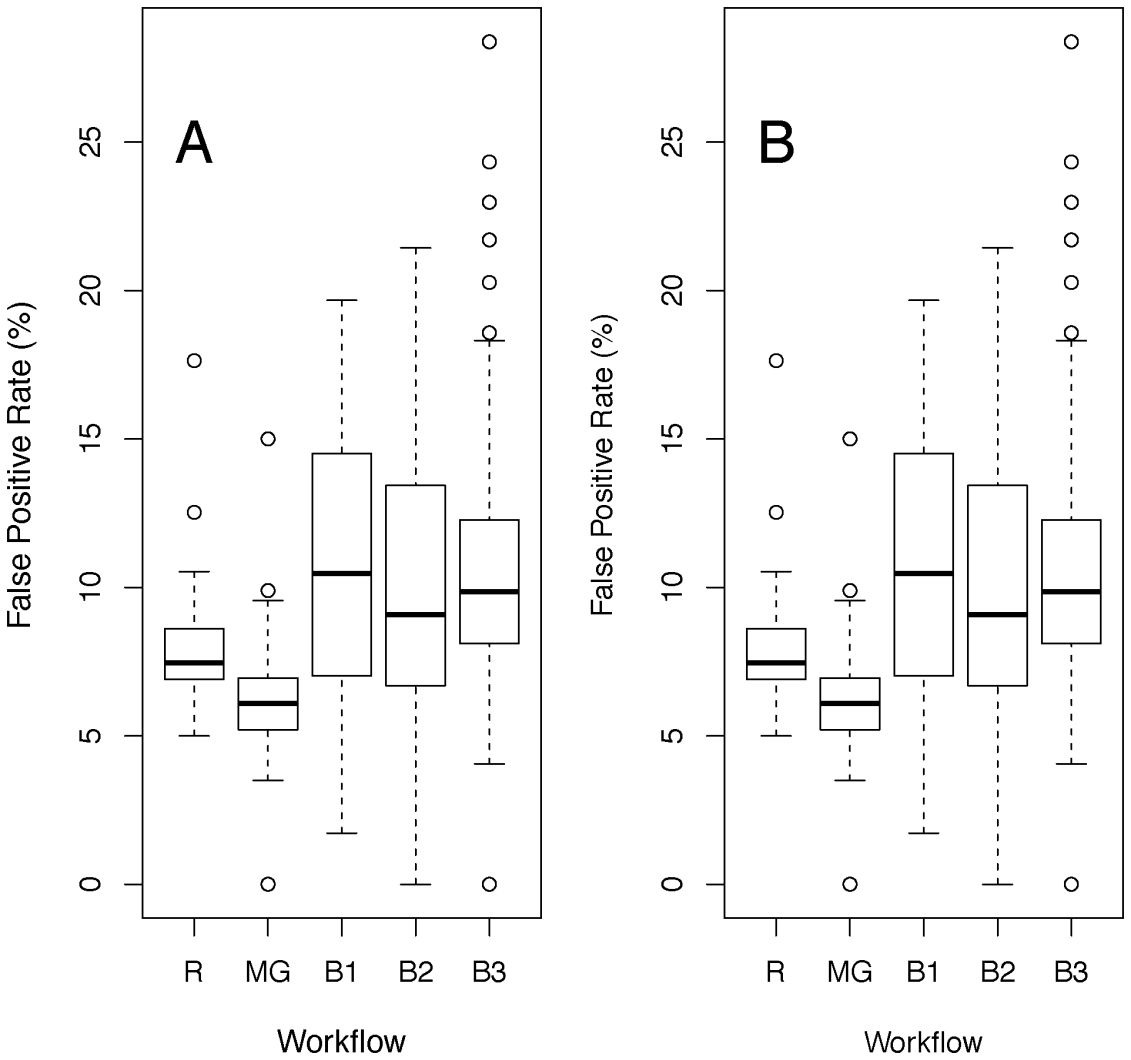
False Positive Rates for resampling control data for various workflows. Shown are FPRs under the null hypothesis of no phenotypic effect, estimated by resampling controls for various workflows, for 70 traits from five assays from the WTSI Mouse Genetics Project (MGP) Select Pipeline. The y-axis shows box-and-whisker plots of the distribution of the FPR, defined as the fraction of resampled datasets significant at the nominal 5% level in a mixed model. A: FPR of the test of genotype effect. B: FPR of the test of sex by genotype interaction. The labels relate to the workflows as defined in table one where MG indicates the Multi-Group, R the Random, B2 the TwoBatch, B1 the OneBatch and B3 the ThreeBatch workflows.

**Figure 6:**
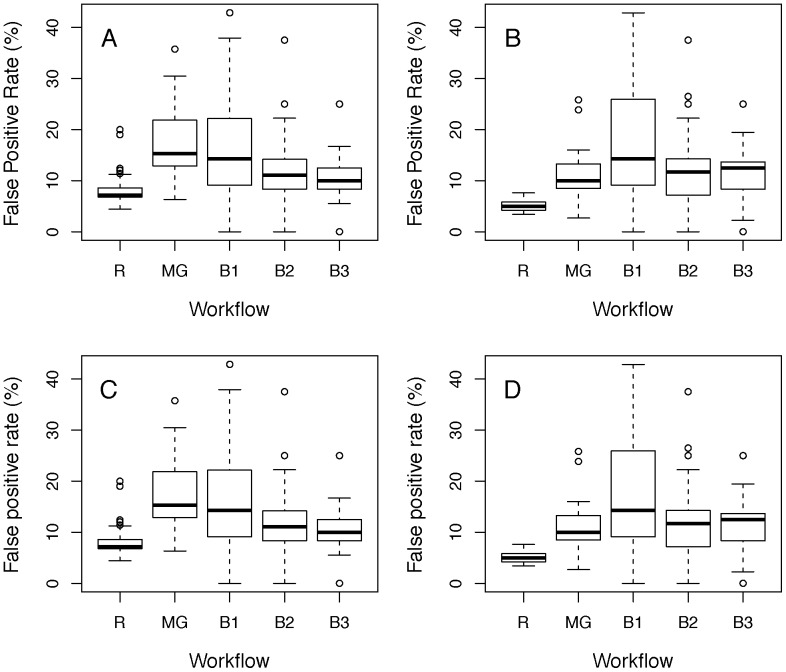
False Positive Rates for other institutes for various workflows. Shown are FPRs under the null hypothesis of no phenotypic effect, estimated by resampling controls from two independent institutes for various workflows. The y-axis’s show box-and-whisker plots of the distribution of the FPR, defined as the fraction of resampled datasets significant at the nominal 5% level in a mixed model. A, B: Resampling results using data from the German Mouse Clinic, where A is the FPR for genotype effect and B is the FPR for genotype-by-sex effect. C, D: Results using control data from Institut Clinique de la Souris, where C is the FPRs for the genotype effect and D the FPRs for the genotype-by-sex effect. The labels relate to the workflows as defined in table one where MG indicates the Multi-Group, R the Random, B2 the TwoBatch, B1 the OneBatch and B3 the ThreeBatch workflows.

As would be expected if there were large fluctuations between batches, *low-batch* and traditional workflows with the MM methodology showed elevated false positive rates for both the genotype and the genotype-by-sex effects (Figure 5). We observed similar behaviour when we resampled control data from other institutes (Figure 6). However, in contrast to the WTSI data, the multi-batch workflow with ICS dataset also had an inflated FPR. ICS has a lower throughput than WTSI and typically uses a concurrent control design. Consequently data are collected less frequently and the dataset available for resampling were smaller. The proportion of variance attributable to batch was higher in the ICS data relative to the WTSI (paired *t*-Test, p = 3.81e-4, df = 47, difference: 6.3±1.3% (mean ± standard error of the mean), possibly due to larger time intervals between batches.

### Resampling of artificially constructed data

**Figure 7:**
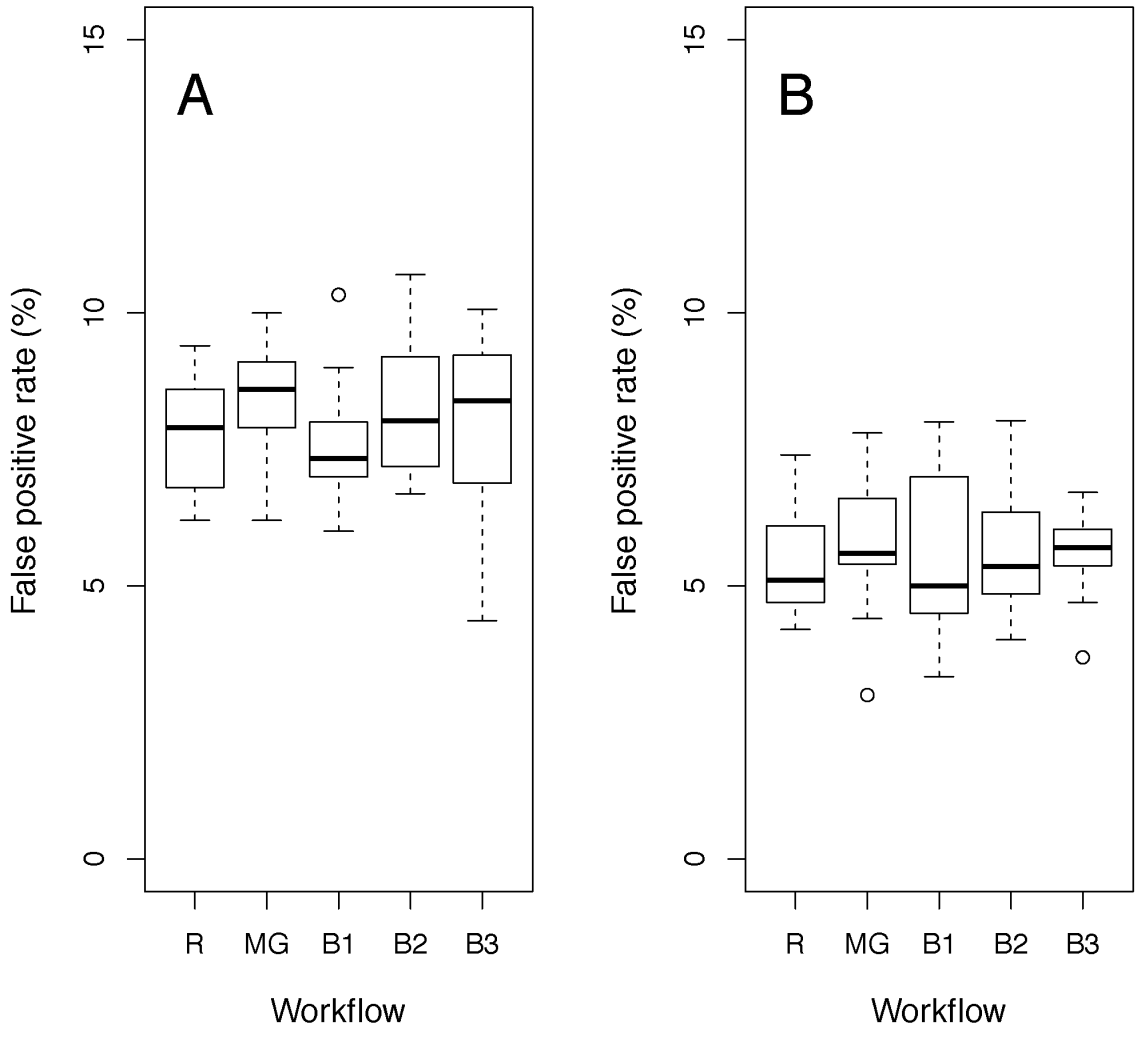
False Positive Rates for simulated data for various workflows. Shown are FPRs under the null hypothesis of no phenotypic effect, estimated by resampling simulated controls. The y-axis’s show box-and-whisker plots of the distribution of the FPR, defined as the fraction of resampled datasets significant at the nominal 5% level in a mixed model. A: FPRs of the test of genotype effect. B: FPRs of the test of genotype-by-sex effect. The labels relate to the workflows as defined in table one where MG indicates the Multi-Group, B2 the TwoBatch, B1 the OneBatch and B3 the ThreeBatch workflows.

To understand the source of the increased FPR of the MM with some workflows, artificial control data were constructed based on the current understanding of variation in the control data. As this knowledge has been used to construct the mixed model, this control data would met the assumptions of the mixed model perfectly. The resampling study was repeated on this data (Figure 7). The distribution of false positive rates became independent of workflow, though was slightly higher than expected, e.g. with an average rate 8% at the 5% significance threshold. The higher FPR is due to the iterative nature of the testing, where the model is optimised for the presence or absence of sexual dimorphism. This introduces a slight bias towards calling differences and hence a higher FPR. As the effect is consistent across workflows and significance thresholds, we can adjust the *p*-value threshold used to achieve the desired FPR (Figure S3).

### Power analysis

**Figure 8:**
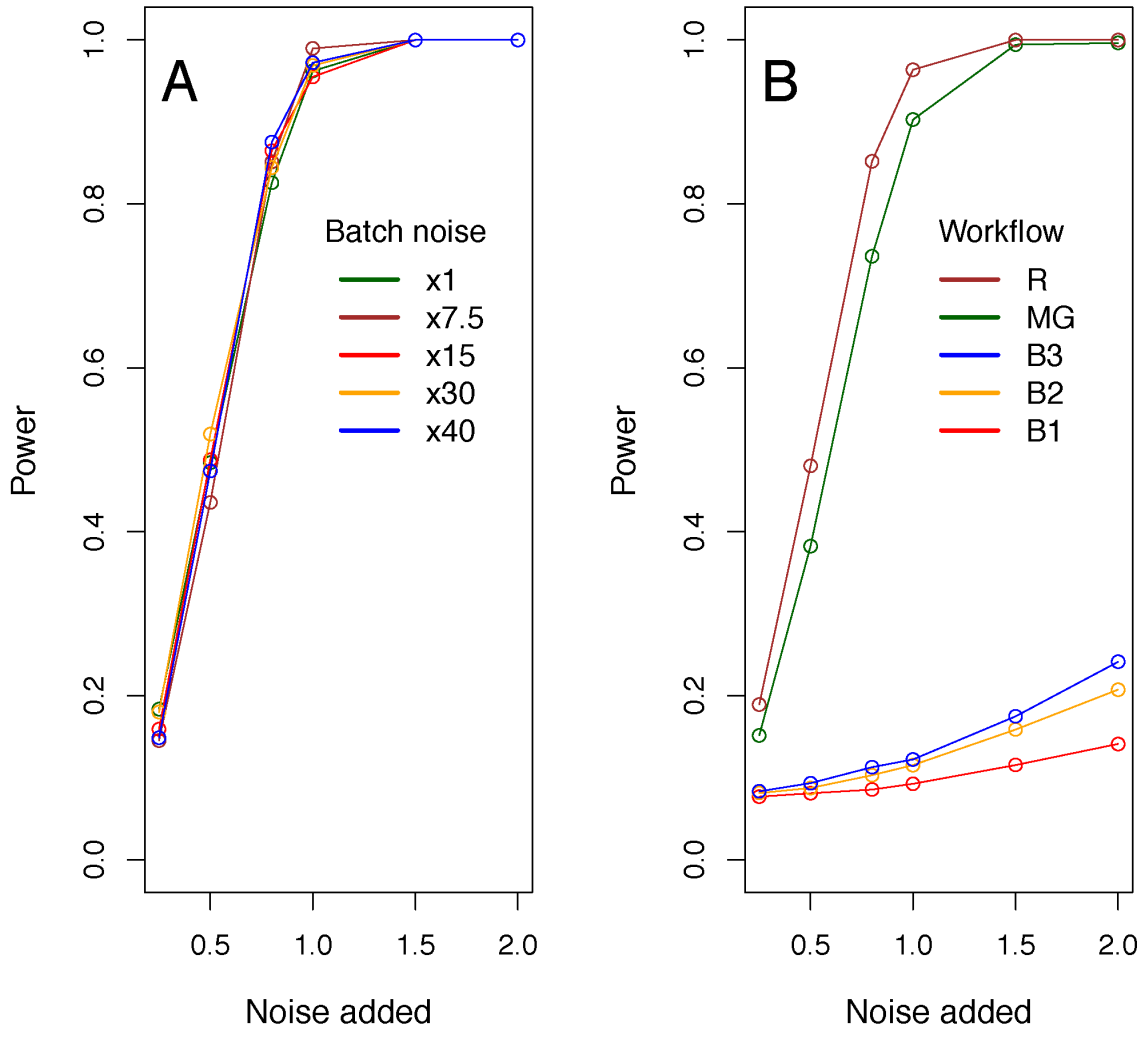
Power analysis - impact of workflow and batch variation. The mixed model methodology sensitivity was assessed with a resampling study on simulated control data where a signal as a proportion of the biological variation was added to construct a knockout group prior to statistical comparison. A: Impact of increasing batch noise, where the noise was a multiplier of 10% of the biological noise. Data shown are the output for simulated bone mineral density trait with a random workflow and a 0.05 significance threshold. B: Impact of workflow. Data shown are the output for the five workflows considered within this manuscript for a 0.05 significance threshold.

For the various workflows where we have seven males and seven female knockout mice, we next considered situations where there was a genuine difference between knockouts and controls by adding an offset (the genotype effect, scaled in units of biological standard deviation) to the phenotypes of the simulated knockout animals, in order to estimate power. As would be expected, as the genotype effect increased, so did the power. This sensitivity was independent of the extent of batch variation (Figure 8a) as the mixed model separates the batch variation from the genotype variation. Also as expected, with more stringent significance thresholds (for example the IMPC analysis pipeline currently uses 0.0001) power decreases (Figure S4). With a random workflow and a significance threshold of 0.05, 80% power is achieved when the genotype effect is equivalent to 0.75*standard deviation units, whilst with the significance threshold of 0.0001, 80% power is achieved when the genotype effect is equivalent to 1.35*standard deviation units. The workflow had a significant impact on sensitivity (Figure 8b); sensitivity was higher when knockout mice were phenotyped in more batches.

## Discussion

Understanding the issues surrounding *in vivo* high-throughput mouse phenotyping is critical in order that we understand the genotype-phenotype map. In particular the control of temporal variation is a significant challenge. Its impact depends on whether there are concurrent controls and whether mice for a knockout line are phenotyped in single or multiple batches. This also raises the question of how to analyse the data robustly. These issues are not unique to high throughput phenotyping, because in small-scale studies fertility and cost issues associated with breeding make it difficult to obtain sufficient animals at one point in time. Experiments run over multiple batches have greater generalizability but this means the analysis has to account for the temporal variation appropriately.

Our simulations show that the mixed model methodology has the expected FPR in workflows with many batches of mice (multi-batch workflows) but that the FPR is inflated in *low-batch* and traditional workflows. This is the case across all five assays tested, selected to represent the breadth of phenotyping tests in different centres. In contrast, our simulations with artificially constructed data showed that the FPR was independent of workflow and similar to that with real control data in random or *multi-batch* workflows, suggesting MM models real data well in these workflows. The discrepancy between real control and simulated data in *low-batch* workflows shows that phenotyping mice in only a few batches is likely to increase the FPR. We suggest that extreme environmental fluctuations, leading to atypical control data, caused significant phenotype calls in *low-batch/traditional* workflows as the model assumed the difference between controls and KO animals was entirely genotypic.

The MM is sensitive technique for a *multi-batch workflow* with 80% power to detect a genotype effect equivalent to 0.75–0.87 times the population standard deviation (depending on exact *multi-batch* implementation) with 7 males and 7 females when compared to a large set of controls with a 5% significance threshold. Power was independent of the size of the batch variance. Power was significantly lower with low-batch and traditional workflow; indicating that in such workflows the MM has poor control of false positives and high rate of false negatives.

Our results suggest that a single statistical analysis pipeline for the IMPC is inappropriate unless the same workflow is used in all centres. Where knockout mice are phenotyped in many small batches, the MM minimises the risk of the genotype effect being confounded by unexpected temporal variation. Alternate analysis strategies may be more appropriate for other workflows if they can model the over-dispersion of temporal variation better. For example, in a workflow comprising a single batch of mice with concurrent controls (traditional workflow), temporal effects are avoided and traditional statistical methods (e.g. student’s *t*-test) are appropriate, though the generalizability of the results are lower. In practice, it is usual to obtain mice in small batches due to fertility or fecundity issues, so another option would be to phenotype the mice with concurrent controls within each batch and analyse the data using a regression method where batch is treated as a fixed effect. This would be a common scenario for secondary phenotyping experiments, to follow up lines of interest. However as the number of knockout mice are typically low for a line, we suggest that at least two mice for each genotype in each batch are phenotyped to obtain accurate estimates.

Our findings have implications not only for *in-vivo* mouse studies, but also for general phenotyping experiments, including cell line studies, that are subject to batch variation. The statistical analysis of high-throughput studies is an important but relatively neglected field. It might be thought that because the comparisons involved are simple (case *vs* control) so is the analysis. However, we have shown here that this is not the case, because it is rarely possible to control the environment completely. High-throughput studies that ignore the effect of environment on the experimental design are therefore liable to produce unreliable conclusions.

## Supporting Information

Figure S1 **Diagrammatic representation of the various sampling strategies tested to mimic various workflows.**
(DOCX)

Figure S2 **The false positive rate observed with resampling real control data at WTSI for various workflows.**
(DOCX)

Figure S3 **The false positive rate observed with resampling simulated control data for various significant thresholds.**
(DOCX)

Figure S4 **The statistical power of the mixed model methodology.**
(DOCX)

Table S1 **Number of iterations of resampling achieved for various studies.**
(DOCX)

Table S2 **Resampling p value profile with various workflows.**
(DOCX)

Code S1 **Construction of artificial phenotyping data using R.**
(DOCX)

Methods S1 **Supplementary Methods.**
(DOCX)
