# Supplementary material for: Impact of Temporal Variation on Design and Analysis of Mouse Knockout Phenotyping Studies
Source: PLoS One. 2014 Oct 24;9(10):e111239. doi: 10.1371/journal.pone.0111239 (PMC4208881; doi:10.1371/journal.pone.0111239)

Figure S1

**Diagrammatic representation of the various sampling strategies tested to mimic various workflows.**

Shown is how mice were selected from the control dataset for relabeling as knockout mice for the various workflows.

A: OneBatch


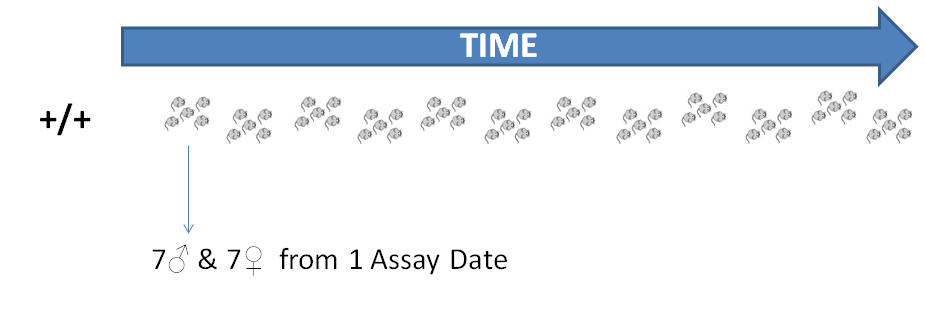


B: TwoBatch


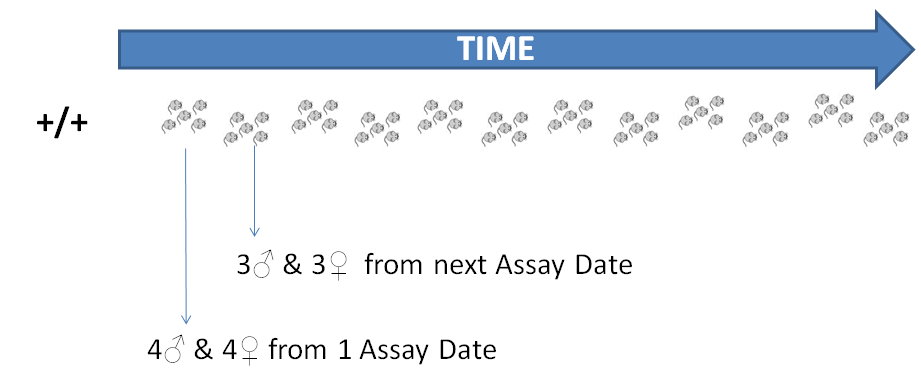


C: ThreeBatch


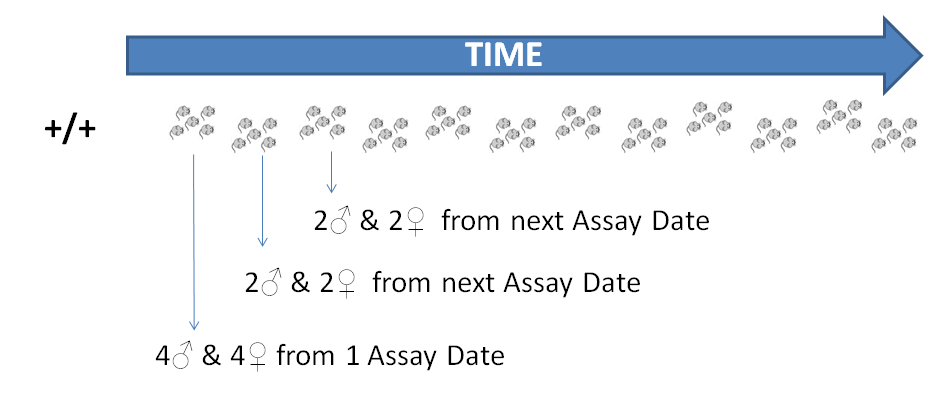


D: Multi-Group


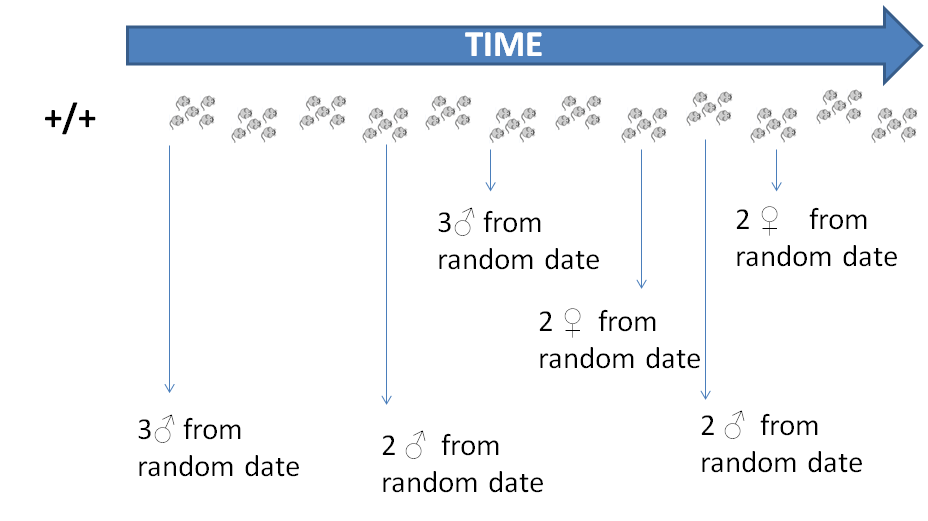


E: Random


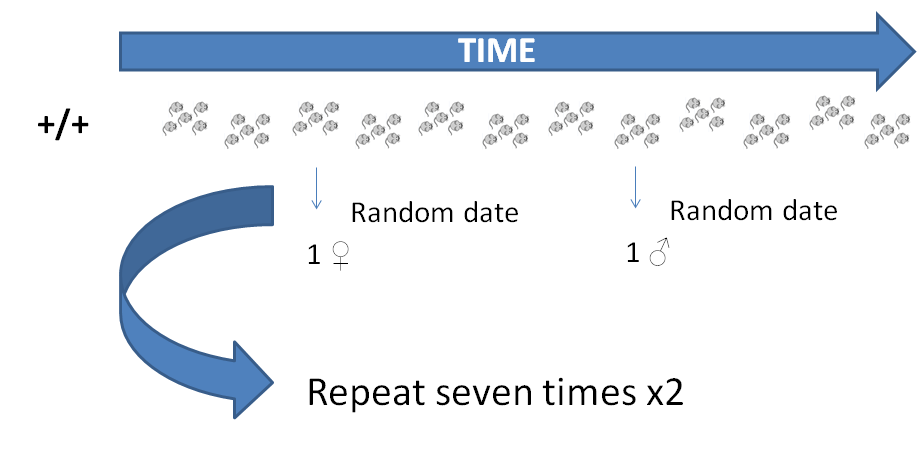

Supplement: Figure S1 — Diagrammatic representation of the various sampling strategies tested to mimic various workflows. (DOCX) [file pone.0111239.s001.docx]
