# Supplementary material for: Impact of Temporal Variation on Design and Analysis of Mouse Knockout Phenotyping Studies
Source: PLoS One. 2014 Oct 24;9(10):e111239. doi: 10.1371/journal.pone.0111239 (PMC4208881; doi:10.1371/journal.pone.0111239)
Supplement: Figure S2 — The false positive rate observed with resampling real control data at WTSI for various workflows. (DOCX) [file pone.0111239.s002.docx]

**Figure S2: The false positive rate observed with resampling real control data at WTSI for various workflows**

The variation in false positive rate for the assessment of the genotype effect (A) and for genotype-by-sex effect (B) for different workflows for various significance thresholds when WTSI control data for five assays was resampled. The impact of workflow is independent of the significant threshold chosen.

A:

B:
