# Supplementary material for: Impact of Temporal Variation on Design and Analysis of Mouse Knockout Phenotyping Studies
Source: PLoS One. 2014 Oct 24;9(10):e111239. doi: 10.1371/journal.pone.0111239 (PMC4208881; doi:10.1371/journal.pone.0111239)
Supplement: Figure S3 — The false positive rate observed with resampling simulated control data for various significant thresholds. (DOCX) [file pone.0111239.s003.docx]

**Supplementary Figure 3: The false positive rate observed with resampling simulated control data for various significant thresholds**

The variation in false positive rate for the assessment of the genotype effect (A) and for genotype-by-sex effect (B) for different workflows for various significance thresholds when simulated control data was resampled. For each effect, the relationship between the FPR and significance threshold was quantified and output shown in the table with each graph. Using this relationship, an adjusted p-value threshold can be calculated to achieve the desired FPR. For example, for the genotype effect a 5% FPR would be achieved using a significance threshold of 0.03. An equivalent line indicates the ideal false positive rate (i.e. 5% at 0.05 threshold).

| Model | FPR ~Significant Threshold | | | |
| --- | --- | --- | --- | --- |
| Coefficients | Estimate | Std. Error | T value | Pr(>\|t\|) |
| Intercept | 0.26856 | 0.0504 | 5.33 | 1.12e-5 |
| Significant threshold | 156.856 | 2.1639 | 72.49 | <2e-16 |
| R-Squared | 0.9946 |  |  |  |

A

| Model | FPR ~Significant Threshold | | | |
| --- | --- | --- | --- | --- |
| Coefficients | Estimate | Std. Error | t value | Pr(>\|t\|) |
| Intercept | 0.202 | 0.0303 | 6.64 | 3.32e-7 |
| Significant threshold | 109.4 | 1.3052 | 83.83 | <2e-16 |
| R-Squared | 0.9946 |  |  |  |

B
