# Supplementary material for: Impact of Temporal Variation on Design and Analysis of Mouse Knockout Phenotyping Studies
Source: PLoS One. 2014 Oct 24;9(10):e111239. doi: 10.1371/journal.pone.0111239 (PMC4208881; doi:10.1371/journal.pone.0111239)

**Figure S4: The power of the mixed model methodology**

Simulated control data was sampled to give a set of knockout mice, to these a treatment effect was added, and the resulting dataset tested with the mixed model methodology. The process was repeated over 500 iterations for each variable and scenario. A: Sensitivity for various variables at the 0.05 threshold when using a random workflow. B: Sensitivity for various variables at the 0.0001 threshold when using a random workflow.

A.


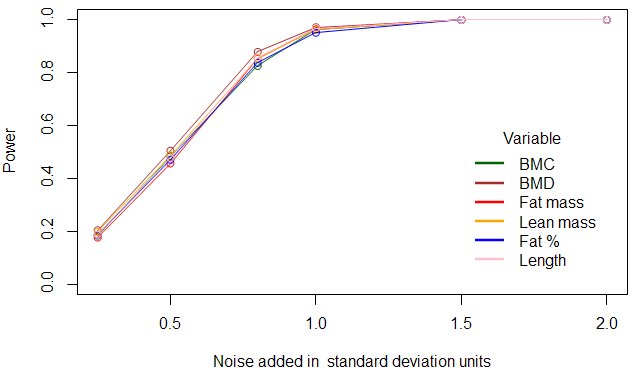


B.


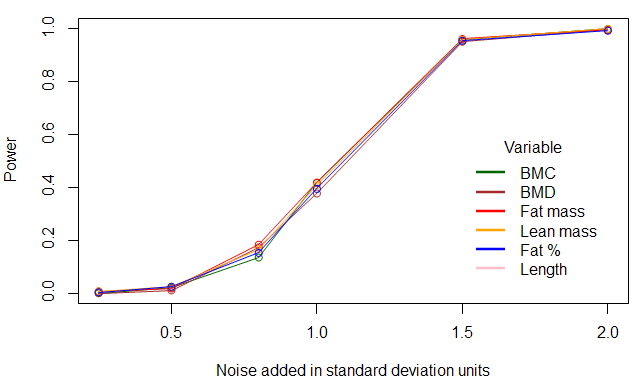

Supplement: Figure S4 — The statistical power of the mixed model methodology. (DOCX) [file pone.0111239.s004.docx]
