# Supplementary material for: Impact of Temporal Variation on Design and Analysis of Mouse Knockout Phenotyping Studies
Source: PLoS One. 2014 Oct 24;9(10):e111239. doi: 10.1371/journal.pone.0111239 (PMC4208881; doi:10.1371/journal.pone.0111239)
Supplement: Table S1 — Number of iterations of resampling achieved for various studies. (DOCX) [file pone.0111239.s005.docx]

**Table S1: Number of iterations of resampling for various studies**

The resampling process built a knockout dataset from control data for an assay by selecting mice and relabeling them to knockout, as multiple variables are measured within an assay , the final output depends on the number of times datasets were build but also the number of dependent variables within each dataset. The number of iterations, particularly for the OneBatch, TwoBatch and ThreeBatch workflow, is limited by total sample size and also number of measures within each batch.

| Institute | Workflow | **Assay** | **Iterations** | **Number variables in each iterations** |
| --- | --- | --- | --- | --- |
| WTSI | Random | Dual-energy X-ray absorptiometry | 1500 | 7 |
|  |  | Clinical chemistry | 1000 | 25 |
|  |  | [Peripheral](https://mouse.internal.sanger.ac.uk/MouseGeneticsCentral/view_mouse_line_test.do?testTemplateId=50&mouseLineId=747) blood leukocytes | 1000 | 12 |
|  |  | Open field | 1000 | 16 |
|  |  | Haematology | 1000 | 10 |
|  | Multi-Group | Dual-energy X-ray absorptiometry | 1500 | 7 |
|  |  | Clinical chemistry | 900 | 25 |
|  |  | [Peripheral](https://mouse.internal.sanger.ac.uk/MouseGeneticsCentral/view_mouse_line_test.do?testTemplateId=50&mouseLineId=747) blood leukocytes | 1000 | 12 |
|  |  | Open field | 1000 | 16 |
|  |  | Haematology | 1000 | 10 |
|  | OneBatch | Dual-energy X-ray absorptiometry | 63 | 7 |
|  |  | Clinical chemistry | 62 | 25 |
|  |  | [Peripheral](https://mouse.internal.sanger.ac.uk/MouseGeneticsCentral/view_mouse_line_test.do?testTemplateId=50&mouseLineId=747) blood leukocytes | 57 | 12 |
|  |  | Open field | 58 | 16 |
|  |  | Haematology | 66 | 10 |
|  | TwoBatch | Dual-energy X-ray absorptiometry | 67 | 7 |
|  |  | Clinical chemistry | 67 | 25 |
|  |  | [Peripheral](https://mouse.internal.sanger.ac.uk/MouseGeneticsCentral/view_mouse_line_test.do?testTemplateId=50&mouseLineId=747) blood leukocytes | 64 | 12 |
|  |  | Open field | 66 | 16 |
|  |  | Haematology | 70 | 10 |
|  | ThreeBatch | Dual-energy X-ray absorptiometry | 74 | 7 |
|  |  | Clinical chemistry | 74 | 25 |
|  |  | [Peripheral](https://mouse.internal.sanger.ac.uk/MouseGeneticsCentral/view_mouse_line_test.do?testTemplateId=50&mouseLineId=747) blood leukocytes | 71 | 12 |
|  |  | Open field | 106 | 16 |
|  |  | Haematology | 74 | 10 |
| Institut Clinique de la Souris | Random | Dual-energy X-ray absorptiometry | 500 | 8 |
|  |  | Clinical chemistry -fasted | 500 | 5 |
|  |  | Clinical chemistry – non fasted | 500 | 17 |
|  |  | [Peripheral](https://mouse.internal.sanger.ac.uk/MouseGeneticsCentral/view_mouse_line_test.do?testTemplateId=50&mouseLineId=747) blood leukocytes | 500 | 9 |
|  |  | Open field | 500 | 17 |
|  |  | Haematology | 500 | 8 |
|  | Multi-Group | Dual-energy X-ray absorptiometry | 300 | 8 |
|  |  | Clinical chemistry -fasted | 300 | 5 |
|  |  | Clinical chemistry – non fasted | 300 | 17 |
|  |  | [Peripheral](https://mouse.internal.sanger.ac.uk/MouseGeneticsCentral/view_mouse_line_test.do?testTemplateId=50&mouseLineId=747) blood leukocytes | 300 | 9 |
|  |  | Open field | 300 | 17 |
|  |  | Haematology | 150 | 8 |
|  | OneBatch | Dual-energy X-ray absorptiometry | 34 | 8 |
|  |  | Clinical chemistry -fasted | 14 | 5 |
|  |  | Clinical chemistry – non fasted | 66 | 17 |
|  |  | [Peripheral](https://mouse.internal.sanger.ac.uk/MouseGeneticsCentral/view_mouse_line_test.do?testTemplateId=50&mouseLineId=747) blood leukocytes | 52 | 9 |
|  |  | Open field | 48 | 17 |
|  |  | Haematology | 58 | 8 |
|  | TwoBatch | Dual-energy X-ray absorptiometry | 60 | 8 |
|  |  | Clinical chemistry -fasted | 8 | 5 |
|  |  | Clinical chemistry – non fasted | 36 | 17 |
|  |  | [Peripheral](https://mouse.internal.sanger.ac.uk/MouseGeneticsCentral/view_mouse_line_test.do?testTemplateId=50&mouseLineId=747) blood leukocytes | 36 | 9 |
|  |  | Open field | 56 | 17 |
|  |  | Haematology | 34 | 8 |
|  | ThreeBatch | Dual-energy X-ray absorptiometry | 60 | 8 |
|  |  | Clinical chemistry -fasted | 44 | 5 |
|  |  | Clinical chemistry – non fasted | 34 | 17 |
|  |  | [Peripheral](https://mouse.internal.sanger.ac.uk/MouseGeneticsCentral/view_mouse_line_test.do?testTemplateId=50&mouseLineId=747) blood leukocytes | 8 | 9 |
|  |  | Open field | 36 | 17 |
|  |  | Haematology | 36 | 8 |
| German Mouse Clinic | Random | Dual-energy X-ray absorptiometry | 500 | 9 |
|  |  | Clinical chemistry | 500 | 22 |
|  |  | [Peripheral](https://mouse.internal.sanger.ac.uk/MouseGeneticsCentral/view_mouse_line_test.do?testTemplateId=50&mouseLineId=747) blood leukocytes | Insufficient data | |
|  |  | Open field | 500 | 14 |
|  |  | Haematology | 500 | 13 |
|  | Multi-Group | Dual-energy X-ray absorptiometry | 300 | 9 |
|  |  | Clinical chemistry | 300 | 22 |
|  |  | [Peripheral](https://mouse.internal.sanger.ac.uk/MouseGeneticsCentral/view_mouse_line_test.do?testTemplateId=50&mouseLineId=747) blood leukocytes | Insufficient data | |
|  |  | Open field | 300 | 14 |
|  |  | Haematology | 300 | 13 |
|  | OneBatch | Dual-energy X-ray absorptiometry | 6 | 9 |
|  |  | Clinical chemistry | 6 | 22 |
|  |  | [Peripheral](https://mouse.internal.sanger.ac.uk/MouseGeneticsCentral/view_mouse_line_test.do?testTemplateId=50&mouseLineId=747) blood leukocytes | Insufficient data | |
|  |  | Open field | 7 | 14 |
|  |  | Haematology | 7 | 13 |
|  | TwoBatch | Dual-energy X-ray absorptiometry | 9 | 9 |
|  |  | Clinical chemistry | 14 | 22 |
|  |  | [Peripheral](https://mouse.internal.sanger.ac.uk/MouseGeneticsCentral/view_mouse_line_test.do?testTemplateId=50&mouseLineId=747) blood leukocytes | Insufficient data | |
|  |  | Open field | 17 | 14 |
|  |  | Haematology | 19 | 13 |
|  | ThreeBatch | Dual-energy X-ray absorptiometry | 9 | 9 |
|  |  | Clinical chemistry | 17 | 22 |
|  |  | [Peripheral](https://mouse.internal.sanger.ac.uk/MouseGeneticsCentral/view_mouse_line_test.do?testTemplateId=50&mouseLineId=747) blood leukocytes | Insufficient data | |
|  |  | Open field | 17 | 14 |
|  |  | Haematology | 19 | 13 |
