# Supplementary material for: Impact of Temporal Variation on Design and Analysis of Mouse Knockout Phenotyping Studies
Source: PLoS One. 2014 Oct 24;9(10):e111239. doi: 10.1371/journal.pone.0111239 (PMC4208881; doi:10.1371/journal.pone.0111239)
Supplement: Table S2 — Resampling p value profile with various workflows. (DOCX) [file pone.0111239.s006.docx]

**Table S2: Resampling p value profile with various workflows**

The p value distribution seen in the resampling study where mice were sampled from the control data and then compared back to the remaining data for various workflows that can be implemented. The data shown is the results for WTSI Mouse GP control data for five assays. For random and multi-group workflow the resampling iterations was set at 1000+. The number of iterations for all remaining workflows is limited by the dataset structure (e.g. number of batches with 7 male and 7 female mice).

| Workflow | p value distribution for genotype test | p value distribution for genotype-by-sex test |
| --- | --- | --- |
| Random | 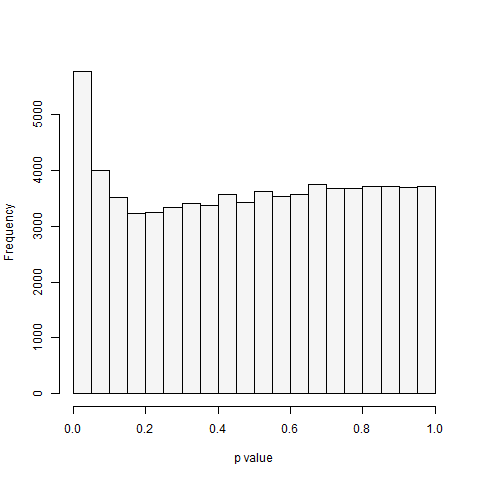 | 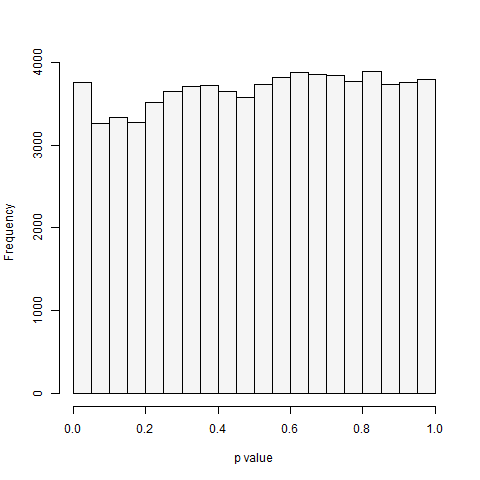 |
| Multi-Group | 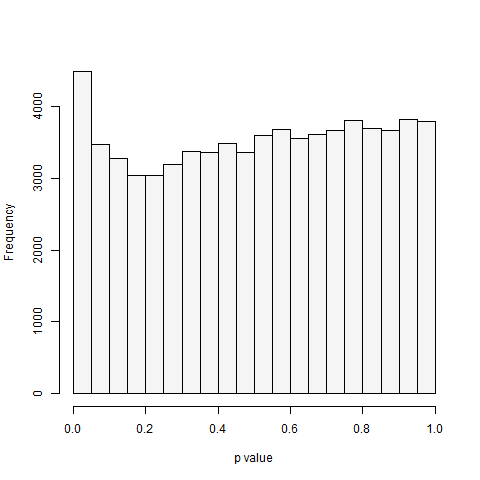 | 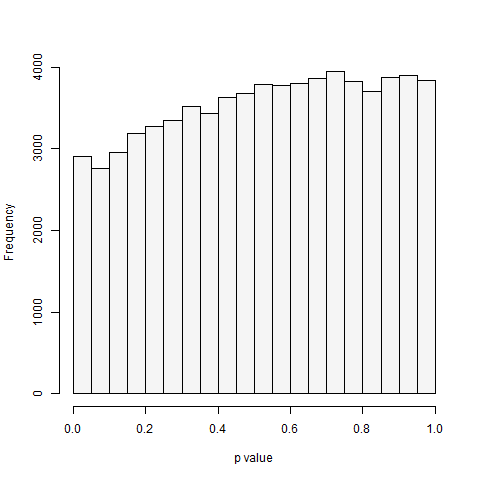 |
| OneBatch | 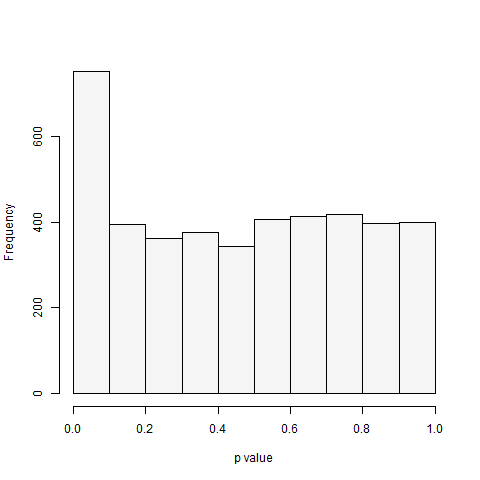 | 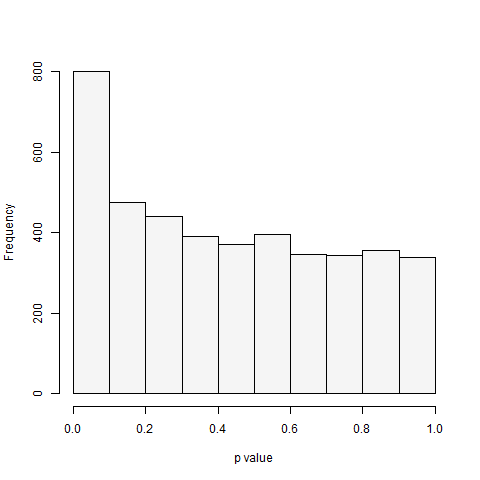 |
| TwoBatch | 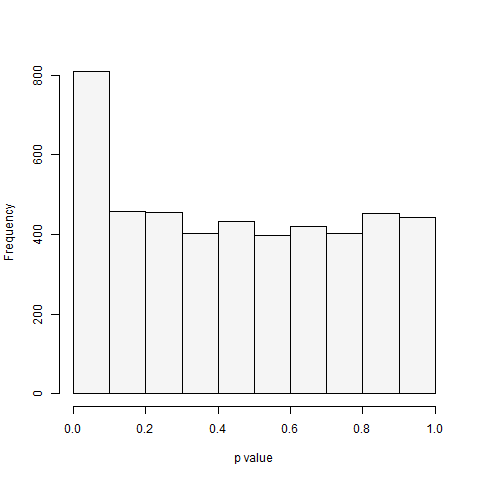 | 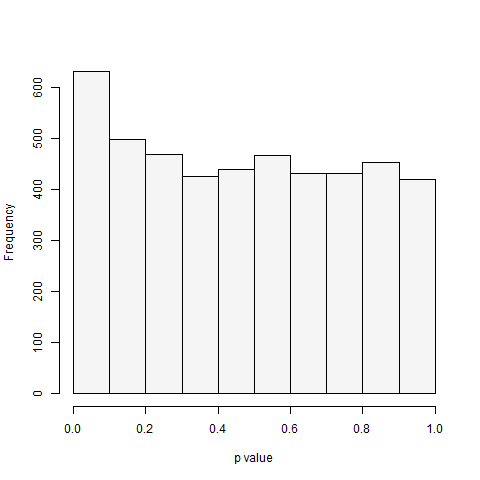 |
| ThreeBatch | 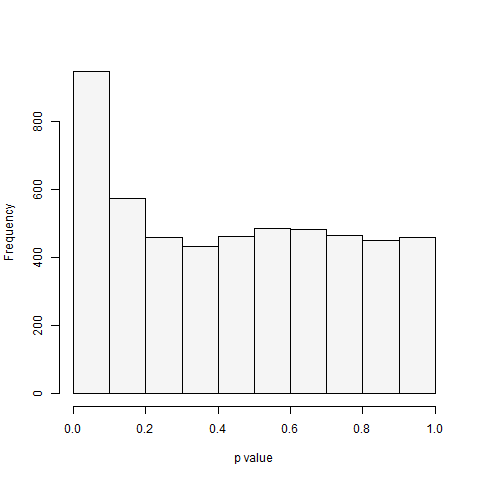 | 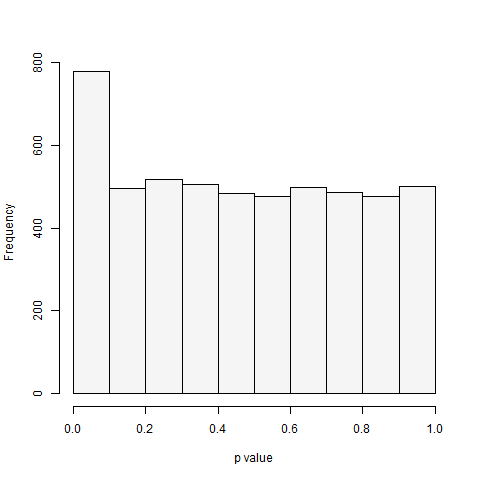 |
