# Supplementary material for: Impact of Temporal Variation on Design and Analysis of Mouse Knockout Phenotyping Studies
Source: PLoS One. 2014 Oct 24;9(10):e111239. doi: 10.1371/journal.pone.0111239 (PMC4208881; doi:10.1371/journal.pone.0111239)
Supplement: Code S1 — Construction of artificial phenotyping data using R. (DOCX) [file pone.0111239.s007.docx]

**Code S1: Construction of artificial phenotyping data**

MockDataSet2 **<-** **function**(NoAssayDates=300, VariableMean, VariableSD, BatchSD, MaleEffect=1){

numberBatches=c(1:NoAssayDates)

BatchEffect=rnorm(n=NoAssayDates, mean=0, sd=BatchSD)

ToDo=data.frame(Index=numberBatches, ImpactBatch=BatchEffect)

results=c()

**for** (bob **in** numberBatches) {

df=subset(x=ToDo, Index==bob)

DependentVariable_Female=rnorm(n=7, mean=(VariableMean+df$ImpactBatch), sd=VariableSD)

DependentVariable_Male=rnorm(n=7, mean=(VariableMean+df$ImpactBatch+MaleEffect), sd=VariableSD)

DependentVariable=c(DependentVariable_Female, DependentVariable_Male)

Gender=c(rep.int("Female", times=7), rep.int("Male", times=7))

Genotype=rep.int("+/+", times=14)

Assay.Date=rep.int(x=paste(bob, "date", sep="_"), times=14)

ThisBatchResults=data.frame(col1=DependentVariable, col2=Assay.Date, col3=Genotype, col4=Gender)

results=rbind(results, ThisBatchResults)

}

names(results)=c("testV", "Assay.Date", "Genotype", "Gender")

results$Assay.Date=factor(results$Assay.Date)

return(results)

}

DependentV1=MockDataSet2(NoAssayDates=300, VariableMean=7.0, VariableSD=1.5, BatchSD=0.5, MaleEffect=1)

DependentV2=MockDataSet2(NoAssayDates=300, VariableMean=6.0, VariableSD=1.3, BatchSD=0.4, MaleEffect=1)

DependentV3=MockDataSet2(NoAssayDates=300, VariableMean=9.0, VariableSD=1.0, BatchSD=0.5, MaleEffect=1)

output=MockDataSet2(NoAssayDates=300, VariableMean=0.02, VariableSD=0.005, BatchSD=0.001, MaleEffect=0.01)

output2=MockDataSet2(NoAssayDates=300, VariableMean=2, VariableSD=0.5, BatchSD=0.005, MaleEffect=0.5)

output3=MockDataSet2(NoAssayDates=300, VariableMean=600, VariableSD=50, BatchSD=5, MaleEffect=100)

output4=MockDataSet2(NoAssayDates=300, VariableMean=200, VariableSD=25, BatchSD=10, MaleEffect=40)

output5=MockDataSet2(NoAssayDates=300, VariableMean=100, VariableSD=20, BatchSD=5, MaleEffect=15)

output6=MockDataSet2(NoAssayDates=300, VariableMean=300, VariableSD=15, BatchSD=5, MaleEffect=50)

output7=MockDataSet2(NoAssayDates=300, VariableMean=50, VariableSD=10, BatchSD=1, MaleEffect=10)

output8=MockDataSet2(NoAssayDates=300, VariableMean=2, VariableSD=0.25, BatchSD=0.05, MaleEffect=0.5)

output9=MockDataSet2(NoAssayDates=300, VariableMean=1, VariableSD=0.04, BatchSD=0.005, MaleEffect=0.5)

output10=MockDataSet2(NoAssayDates=300, VariableMean=40, VariableSD=4, BatchSD=2, MaleEffect=8)

output11=MockDataSet2(NoAssayDates=300, VariableMean=5, VariableSD=0.25, BatchSD=0.1, MaleEffect=0.5)

output12=MockDataSet2(NoAssayDates=300, VariableMean=25, VariableSD=3, BatchSD=1, MaleEffect=3)
