# Supplementary material for: Impact of Temporal Variation on Design and Analysis of Mouse Knockout Phenotyping Studies
Source: PLoS One. 2014 Oct 24;9(10):e111239. doi: 10.1371/journal.pone.0111239 (PMC4208881; doi:10.1371/journal.pone.0111239)
Supplement: Methods S1 — Supplementary Methods. (DOCX) [file pone.0111239.s008.docx]

**Supplementary - Methods**

**Datasets:**

Wellcome Trust Sanger Institute (WTSI) control datasets for five different assays were obtained from the standardised WTSI MGP pipeline. This pipeline ran from August 2009 until February 2012 and collected control data with seven males and females weekly. Data were collected for the C57BL/6N (MGI:2159965) background except for open field assay where C57BL/6Brd-*Tyr^c-Brd^*;C57BL/6Dnk;C57BL/6N (MGI:5446362) background provided a larger control dataset for the study (Table 1).

| Assay | Assay dates | Number mice | Number traits monitored | Genetic background |
| --- | --- | --- | --- | --- |
| Clinical chemistry | 108 | 1300 | 27 | C57BL/6N |
| Dual-energy X-ray absorptiometry | 110 | 1307 | 7 | C57BL/6N |
| Open field | 136 | 1481 | 16 | C57BL/6Brd-Tyr^cBrd^; C57BL/6Dnk; C57BL/6N |
| [Peripheral](https://mouse.internal.sanger.ac.uk/MouseGeneticsCentral/view_mouse_line_test.do?testTemplateId=50&mouseLineId=747) blood leukocytes | 93 | 1184 | 10 | C57BL/6N |
| Haematology | 109 | 1325 | 10 | C57BL/6N |

Table 1: Information on the various WTSI dataset compositions.

For the study of Institut Clinique de la Souris (ICS) data (<http://www.ics-mci.fr/en/departments/phenotyping/>), five assays of control data for the genetic background C57BL/6NTac (MGI:2164831) obtained for the standardised EUMODIC Pipeline was downloaded from the International Mouse Phenotyping Consortium Web Portal ([Koscielny, et al., 2013](#_ENREF_3)) by accessing data from the <http://www.europhenome.org/> database. ICS data were generated using the standard SOPs available at <https://www.mousephenotype.org/impress>.

For the study of German Mouse Clinic (GMC) data (<http://www.mouseclinic.de/>) four assays (OF, HAEM, DEXA and CC) of control data for the genetic background C57BL/6NTac (MGI:2164831) were provided.

**Housing and husbandry:**

Housing conditions at the Wellcome Trust Sanger Institute: Mice were maintained in a specific pathogen free unit on a 12hr light: 12hr dark cycle with lights off at 7:30pm and no twilight period. The ambient temperature was 21 ± 2^o^C and the humidity was 55 ± 10%. Mice were housed for phenotyping using a stocking density of 3-5 mice per cage (overall dimensions of caging: (L x W x H) 365 x 207 x 140mm, floor area 530cm^2^) in individually ventilated caging (Tecniplast Seal Safe1284L) receiving 60 air changes per hour. In addition to Aspen bedding substrate, standard environmental enrichment of two nestlets, a cardboard Fun Tunnel and three wooden chew blocks was provided. Mice were given water and diet *ad libitum*. For those mice on the MGP pipeline, at 4 weeks of age, mice were transferred from Mouse Breeders Diet (Lab Diets, 5021-3) to a high fat (21.4% fat by crude content; 42% calories provided by fat) dietary challenge (Special Diet Services, Western RD 829100). For those mice on the MGP select pipeline, presented in the *Expi^tm1a(KOMP)Wtsi^* study, mice were fed on Mouse Breeders Diet (Lab Diets, 5021-3).

Housing conditions at the German Mouse Clinic: Mice were maintained in a specific pathogen free unit on a 12hr light: 12hr dark cycle with lights off at 6pm and no twilight period.  The ambient temperature was 22 ± 2°C and the humidity was 55 ± 10%. Mice were housed for phenotyping using a stocking density of 3-5 mice per cage (overall dimensions of caging: (L x W x H) 365 x 207 x 140mm, floor area 530cm2) in individually ventilated caging (Tecniplast Seal Safe1284L) receiving 60 air changes per hour. Environmental enrichment was provided via a fir and spruce shaving mix with red houses (mouse igloo). Mice were given water and diet ad libitum. The mice were fed on the Altrmon 1314 standard diet.

Housing conditions at the of Institut Clinique de la Souris: Mice were housed in a specific pathogen free unit under a 12-12 light-dark cycle (no twilight period) with controlled temperature (22 ± 2°C) and hygrometry set at 50 ± 10%. Mice were housed for phenotyping using a stocking density of 3-5 mice per cage in individually ventilated caging (Sealsafe GM500, Tecniplast / floor area 530cm2) with 65 air changes per hour and poplar bedding (Lignocell, Germany). No environmental enrichment was provided for the cohort undergoing phenotypic evaluation. Chlorinated water and autoclaved diet (D04, SAFE, France) were provided ad libitum.

**Phenotyping:**

WTSI data were obtained as detailed in ([White, et al., 2013](#_ENREF_4)) following the standard operatoring procedures at <https://www.mousephenotype.org/impress>. Factors thought to affect the variables were standardised as far as possible. Where standardisation was not possible, steps were taken to minimise and randomise to reduce potential bias e.g. the operator effect were managed by passively randomised combined with training and monitoring to minimise the impact. Blinding was not possible within the WTSI environment as the cage cards include genotype information, though with a high throughput environment without defined hypothesis the potential bias is minimised. GMC data were obtained according to Gailus-Durner et al. 2005 and Fuchs et al. 2011([Fuchs, et al., 2011](#_ENREF_1); [Gailus-Durner, et al., 2005](#_ENREF_2)).

**Biological example:**

A study on gene knockout mice carrying the *Expi^tm1a(KOMP)Wtsi^* (MGI:4363398) targeted allele which were created by blastocyst injection of targeted ES cells, and bred on the C57BL/6N genetic background as detailed in White *et al* ([White, et al., 2013](#_ENREF_4)).

**Data Access:**

Data used within this manuscript is freely available from the IMPC project portal (see [http://www.mousephenotype.org](http://www.mousephenotype.org/)) as it can access data from the <http://www.europhenome.org/> database. Data can be downloaded via the Experimental data REST API and instructions for using this application program interface are available at https://github.com/mpi2/PhenotypeArchive/wiki/Experimental-data-REST-API

**References:**

Fuchs, H.*, et al.* (2011) Mouse phenotyping, *Methods*, **53**, 120-135.

Gailus-Durner, V.*, et al.* (2005) Introducing the German Mouse Clinic: open access platform for standardized phenotyping, *Nature methods*, **2**, 403-404.

Koscielny, G.*, et al.* (2013) The International Mouse Phenotyping Consortium Web Portal, a unified point of access for knockout mice and related phenotyping data, *Nucleic Acids Res*, **42**.

White, J.K.*, et al.* (2013) Genome-wide Generation and Systematic Phenotyping of Knockout Mice Reveals New Roles for Many Genes, *Cell*, **154**, 452-464.
